# Assessing potential surge of COVID-19 cases and the need for booster vaccine amid emerging SARS-CoV-2 variants in Indonesia: A modelling study from West Java

**DOI:** 10.1016/j.heliyon.2023.e20009

**Published:** 2023-09-09

**Authors:** Nuning Nuraini, Fadiya Nadhilah Soekotjo, Almira Alifia, Kamal Khairudin Sukandar, Bony Wiem Lestari

**Affiliations:** a Department of Mathematics, Institut Teknologi Bandung, Bandung, 40132, Indonesia; b Research Center for Care and Control of Infectious Disease, Universitas Padjadjaran, Bandung, 40161, Indonesia; c Epidemiology Group of COVID-19 Task Force for West Java, Bandung, 40171, Indonesia; d Department of Public Health, Faculty of Medicine, Universitas Padjadjaran, Bandung, 40161, Indonesia; e Department of Internal Medicine, Radboud University Medical Center, 6525, GA, Nijmegen, the Netherlands

**Keywords:** COVID-19 booster vaccine, Mathematical modelling, Vaccine coverage

## Abstract

**Objectives:**

Primary and booster vaccinations are crucial in COVID-19 control. This study aimed to assess the minimum booster coverage to hamper potential surge of COVID-19 cases in 2023 in Indonesia, a low-resource setting country.

**Methods:**

We used a modified SEIR compartment model to assess different scenarios in booster coverage across West Java population: 35%, 50%, and 70%. We fitted the model, then we calculated the potential active cases in 2023 if each scenario was met before 2022 ends. A heat map of predicted cases from various booster coverages and time frames was produced and matched with vaccination rate's function to determine feasible time frames.

**Results:**

A minimum of 70% booster coverage in West Java is needed to reduce 90% of potential COVID-19 cases and avert possible surge in 2023. The booster doses should be distributed before February 2023 to achieve its optimum preventive effect. Delays in achieving minimum booster coverage is acceptable, but higher booster coverage will be required.

**Conclusions:**

For better COVID-19 control in Indonesia, booster vaccination is warranted, as presented by a case study in West Java. Sufficient vaccine supplies, infrastructure, and healthcare workers should be ensured to support a successful booster vaccination program.

## Highlights

• High booster coverage is warranted to prevent COVID-19 surge in 2023.
• Timely booster rollout is critical in COVID-19 control.
• COVID-19 variants and vaccine brands are accommodated in the model.
• Waning of vaccine-induced immunity is considered in the model.

## 1 Introduction

The COVID-19 pandemic has posed an enormous threat globally by straining health system capacities, including in Indonesia. This is particularly evident during the early phase of the pandemic when the active cases were steadily increasing [1]. To address this situation, vaccination programs have been rolled out across the country since early 2021, and by the end of 2021, 50% of Indonesia's population have received primary vaccine doses [2]. In March 2022, although a high peak of active cases was observed, the corresponding number of deaths was significantly lower compared to that of the earlier peak in September 2021 [3].

However, vaccine-induced immunity, similar to natural immunity, may wane over time. This results in a decrease in vaccine effectiveness. While natural immunity could last longer than a year, vaccine-induced immunity is compromised a few months after inoculation [4,5]. A study in Malaysia demonstrated that CoronaVac's effectiveness against COVID-19 infections waned after three to five months in fully vaccinated people and that the protection against ICU admission declined over time as well [6]. A meta-analysis of eighteen studies also concluded that vaccine effectiveness against SARS-CoV-2 infection waned from 83% within the first month post-vaccination completion to 22% at 5 months or longer [7]. Therefore, the effect of initial vaccination programs will decrease, potentially leading to another surge of cases when the virus is still spreading within the population, especially with variants that are identified to be more transmissible.

Administering booster vaccines is a proven way to reinforce vaccine-induced immunity. Studies in Spain and USA have shown that booster shots can bring added protection to the vaccinated population [8,9]. In Indonesia, booster vaccines have been rolled out since January 2022 and have already been received by 24.2% of the Indonesian population by July 2022 [2]. However, the minimum threshold of booster coverage that will significantly protect the population from encountering another COVID-19 peak has not been identified, including the required period to implement the vaccination program to meet that threshold.

Mathematical modelling of infectious diseases has been proven beneficial in providing such information to advise public health interventions. During the COVID-19 pandemic, multiple models have been developed to understand the disease further and formulate relevant actions. Some relied on modified compartmental SIR models to capture the dynamics of COVID-19 spread, later used for modelling-based inference for policymaking [[10], [11], [12]]. In response to vaccine availability issues, others reported their work in studying the impact of imperfect vaccine efficacy in the vaccine rollout strategy specific to certain regions [[13], [14], [15]]. Furthermore, some studies also incorporated the effect of vaccines and mutant viruses into the models [16]. Zarin et al. employed a modified SEQIR model using finite difference and meshless techniques to incorporate population behaviour in predicting COVID-19 spread [17]. Another study used nonlinear weighted least square estimation and global sensitivity analysis to investigate the optimal control measures for the Delta COVID-19 variant in a partially vaccinated population in China [18].

In this study, we aim to use COVID-19 modelling to inform public health stakeholders of West Java, one of the most populous provinces in Indonesia with a high COVID-19 burden, in rolling out booster vaccines to avoid future COVID-19 health system burdens. We used a modified SEIR model which incorporates the characteristics of COVID-19 vaccines, including the waning of vaccine-induced immunity, to predict COVID-19 active cases in different booster coverage levels. This study provides information regarding the expected minimum booster vaccine coverage and recommended vaccination time frame to prevent future surges of COVID-19 in the region.

## 2 Methods

### 2.1 Setting

Confirmed COVID-19 cases in Indonesia had reached 6.2 million cases in July 2022 with estimated deaths of 157 thousand people [3]. About 1.1 million of the cases, around a sixth of the national number, were attributed to West Java province [19]. In 2020, the province is the most populous in Indonesia, with a total number of 48.2 million residents [20].

Vaccination programs have been rolled out by Indonesia's government since mid-January 2021. At least five vaccine brands have been widely used in Indonesia, namely Sinovac, AstraZeneca, Sinopharm, Pfizer, and Moderna. Approximately 72.8% of the Indonesian population have received primary vaccine doses by 31 July 2022, and 24.4% have received additional booster doses [2].

### 2.2 Data sources

In this study, we used time-series data provided by the Provincial Health Office, consisting of data regarding daily active cases, recorded recovery, and deceased cases from the start of the vaccination program, i.e. 13 January 2021, until 31 July 2022 [21]. The number of administered vaccine doses was also gathered to describe the pattern of the primary vaccination program. Data from Our World in Data, that was sourced from GISAID, about the proportion of SARS-CoV-2 variants from Indonesian sequences were used to illustrate current variants spread [22,23].

### 2.3 Mathematical model and parameters

We employed a mathematical model commonly used to describe disease infection which consists of a nonlinear differential system for four compartments: Susceptible (S), Exposed (E), Infected (I), and Recovered (R) groups of individuals. This partition accommodates the effect of a significant latency period which suits the characteristics of SARS-CoV-2 spread in the human population [24]. Susceptible individuals can get exposed to the virus and will be considered infectious after passing the virus incubation period. Infected individuals will end up being recovered and gaining natural immunity, protecting them for a period of time before becoming susceptible again.

We modified the SEIR model to reflect the effect of vaccination, by dividing each compartment into two subgroups: individuals with and without vaccine protection. We allow both recovered unvaccinated and vaccinated individuals to move back to their respective susceptible groups with a waning rate of 1365 [[4], [5], [6]]. Since vaccine-induced immunity also wanes in susceptible vaccinated individuals, this model allows them to move back to the group of susceptible unvaccinated ones. We followed a similar approach of including immunity waning parameter in COVID-19 compartmental model in Indonesia setting that was used in another study [25].

Notably, there are other mathematical models that are frequently used to study COVID-19 situation and interventions, in the form of statistical models (such as ARIMA) and agent-based models. Although well-trained ARIMA models often produce robust results in disease spread forecasting, due to its linear module structure, non-linearity within the model can reduce its accuracy and it is often deemed unsuitable for dynamic situations [26,27]. It would not accommodate West Java's situation which allows susceptible, exposed, infected, and recovered individuals to interact with each other. In contrary, agent-based modeling offers higher flexibility in capturing disease and individual dynamics, but it requires a more extensive techniques of computational mathematics and takes longer to calibrate [28]. Hence, the modified SEIR model was chosen in our study as it is the best method to incorporate both epidemiological and public health perspectives, and it can provide satisfactory results in simple and timely manners [29,30].

**Fig. 1:**
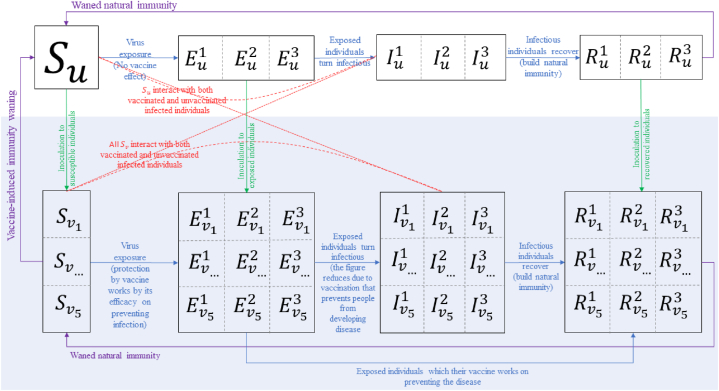
Transmission flow within the modified SEIR (S = Susceptible; E = Exposed; I = Infected; R = Recovered) model with vaccine-based partition of each main compartment. The shaded compartments indicate those with vaccine protection (notated with subscript v), while the ones left unshaded represent groups with no vaccine protection (notated with subscript u). Vaccine inoculations are represented by green lines. No actively infected cases get vaccinated. The red lines show that the susceptible groups can be exposed to SARS-CoV-2 from the infected groups, regardless of the vaccination status. The detailed mathematical model for this flow diagram is given in Appendix 1.

In our model, we consider there are three groups of SARS-CoV-2 variants (n=3) based on their responses to specific vaccine brands. The first group comprises the ancestral virus and alpha variant, the second one includes beta, gamma, and delta variants, while the third one represents only the omicron variant [31]. At the same time, there are five vaccine brands currently used in West Java [2]. To accommodate this information in the model, we split each compartment into subgroups reflecting the spread of COVID-19 variants in the population with different vaccine brands (Fig. 1). Further explanations about the parameters and assumptions of the model depicted in Fig. 1 are given in Appendix 1.

Some parameters in the model were assumed to be constant over time, meaning that aspects like virus mutation or governmental intervention do not significantly affect those parameters. The values of these parameters were derived from relevant references. This group of parameters covers incubation rate [32,33], recovery rate [33], natural and vaccine-induced immunity waning rate [4,6], vaccine efficacy at preventing infections and disease (specific to each brand) [31], and proportions of each vaccine brand's delivered doses [2].

Meanwhile, the vaccination and transmission rates were assumed to be able to change over time. The number of administered vaccine doses within certain periods of vaccination programs, which are contained in the information on vaccination rate, may vary over time depending on how fast the government implements vaccinations and tackles challenges. We specified the rate of vaccination to follow a bell-like curve as explained further in Appendix 1. Estimations of the constants were generated from available data using Markov Chain Monte Carlo (MCMC) method [34] (Appendix 2). At the same time, the transmissibility of the virus is strongly related to the behavior of the general population, which was assumed to be shifting over time as well. Hence, we seek the best time-series values of the time-dependent parameters that result in a simulation that matches the data. Assumptions and calculations regarding vaccination and transmission rates have been validated by fitting to its relevant data, as presented in Appendix 2.

### 2.4 Booster coverage scenarios

We simulated three different scenarios using the model, by varying the proportion of the population that received booster vaccination by the end of December 2022. Vaccination programs before 31 July 2022 have administered booster vaccines to 30% of West Java's population [35]. We assumed the vaccination rate function had stopped at the time of analysis, with 30% booster coverage on July 31st^,^ 2022. Afterwards, we simulated the additional vaccination rate to restart at that point forward. The first scenario simulates the projections of COVID-19 active cases across West Java if a total of 35% of its population obtained booster shots by the end of December 2022, while the second and third scenarios simulate projections of COVID-19 active cases if a total of 50% and 70% of the population received booster shots, respectively.

### 2.5 Vaccination time frame estimation

We produced a heat map that shows other possible scenarios to estimate the feasible time frame for rolling out a booster vaccination program to effectively hamper future COVID-19 surge of active cases. We did point estimates for the number of predicted active cases on each percentage of booster coverage and on each number of days of administering those doses. That number of predicted active cases was then compared to that of the benchmark population (without increased booster coverage), generating the number we call the predicted fraction of total cases. We also combined the point estimates graph to a logistic function that represents the number of administered booster shots over time, based on previous patterns of vaccination programs, to determine the combinations of booster coverage and vaccination time frame that are considered feasible to be implemented. We anticipated the occurrence of an unfeasible region in the calculation as logistically it is difficult to achieve the desired coverage in a short time. Details of the assumptions and calculations for vaccine rollout assessment are explained in Appendix 3. All modelling works were done in MATLAB R2019b. For reproducibility purposes, the MATLAB code of this study will be available upon request to the corresponding author.

## 3 Results

**Fig. 2:**
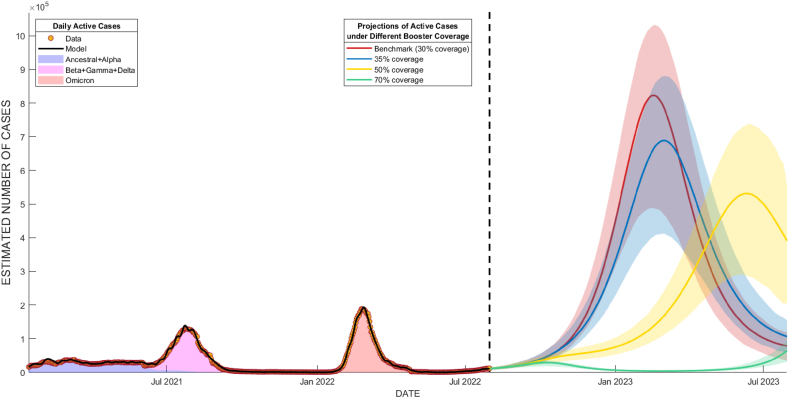
Projections of COVID-19 active cases from early August 2022 to the end of July 2023. The projections were fit to actual daily active cases (black line, from January 2021 to July 2022). The red line represents the benchmark scenario (30% coverage), while the blue, yellow, and green lines illustrate projections for total booster coverage of 35%, 50%, and 70% respectively. Colored areas are the 90% confidence interval for each scenario of the same color.

At the time of analysis, 30% of the West Java population has already received booster shots by 31 July 2022. This projection will act as a benchmark to other simulated scenarios and can be seen as the red line in Fig. 2. Evidently, by the end of 2022, that number only experienced a small increase to 36%, and the daily number of administered booster vaccines in West Java kept getting smaller until early 2023 [35]. This condition was similar to our first scenario, where we assumed the booster coverage would hit a stagnant at 35%.

### 3.1 Booster coverage

Based on our simulated scenarios, to avoid another surge of COVID-19 active cases, West Java needed at least 70% of booster coverage throughout West Java. Full descriptions of modelling scenarios are depicted in Fig. 2. Having lower booster vaccination coverage as simulated in the first and second scenarios, was predicted to cause a surge of COVID-19 cases of 700,000 and 600,000, respectively, as shown in Fig. 2.

### 3.2 Vaccination time frame estimation

Based on the outputs of examined scenarios, an additional 40% of the West Java population should have received their booster shots before January 2023. This means the government should have finalized the booster vaccination within 220 days since July 2022 to achieve the desired outcome in avoiding a possible surge of COVID-19 active cases. Achieving a minimum of 70% of booster vaccination coverage contributed to 90% of preventable cases up to mid-2023. Ideally, 40% of the West Java population should have received their booster shots before mid-February 2023.

**Fig. 3:**
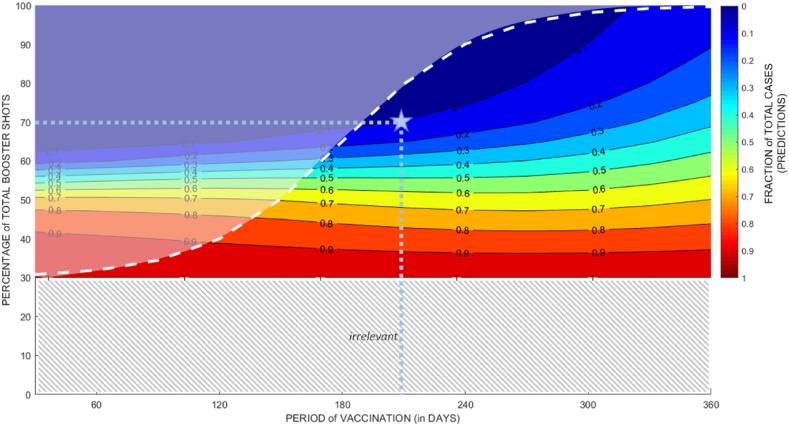
Heat map of the predicted fraction of total cases (number of predicted cases with specific booster coverage and vaccination program duration divided by the total number of predicted cases without any booster intervention). The logistic function (white dashed line) shows the vaccination program's pattern to be rolled out and divides the heat map into two regions. The area above that function is the infeasible region where the combinations of booster coverage and vaccination period are considered infeasible to execute. The meeting point of light blue dotted lines (marked with a blue star) is the recommended combination of booster coverage and vaccination period to avert potential active cases surge.

A more detailed correlation between booster coverage and vaccination delivery time is shown in Fig. 3. The heatmap showed the predicted number of cases with corresponding booster coverage and vaccination delivery time of each point in the area, as a proportion to the predicted number of cases without any booster intervention since the time of analysis, which left the booster coverage constant at 30%. The heatmap showed that higher booster coverage and faster booster delivery led to fewer COVID-19 cases in the future (Fig. 3).

Furthermore, the heatmap was combined with a logistic function (shown in a dashed white line) which illustrates the common vaccination delivery pattern in Indonesia. Planning and initiating new vaccine interventions usually takes time, so delivering a very high number of booster shots in a short time is unlikely. This makes the vaccination program that targets situations beyond this logistic function hard to achieve, hence, we marked the area over the function as an “infeasible region”.

Within the feasible region, it is observable that a total of 70% booster coverage can lead to a 90% reduction of predicted total cases, if delivered within 220 days from 31st July 2022 at the latest. This is the recommended combination because it would bring the highest public health benefit without aiming for higher booster coverage and/or requiring more resource to rollout longer vaccination program. However, a delayed vaccination rollout can be compensated with higher booster coverage to retain public health benefits. Our modelling results suggested that a higher booster coverage of as high as 90% of the West Java population led to a lower number of cases, even if it is administered within 240 days since July 31st, 2022 (shown in Fig. 3).

## 4 Discussions

This study highlights the need for high booster coverage in West Java, with a minimum of 70% coverage administered before February 2023 to avoid the potential surge of COVID-19 active cases in 2023. Beyond that time frame, aiming for higher booster coverage above 90% will provide similar or even better protections for COVID-19 infection control in West Java. Government support to ensure COVID-19 vaccine procurement, adequate supply chain, and distribution is warranted to achieve targeted booster coverage.

Higher booster coverage should be prioritized to provide better infection control for COVID-19 for the community. Our modelling results suggested that delaying vaccination programs or minimizing booster coverage targets would still result in high active cases peak in 2023, making the vaccination rollout to be considered ineffective. Hence, the government should have optimized the delivery of booster shots to the remaining 40% of the population before February 2023. This is in line with another modelling study that recommends administering booster vaccines to global population before winter 2022 or before the influenza season to help mitigate health system stress [36]. Additionally, that study also showed that administering booster doses to all vaccination-eligible people would be more helpful to protect vulnerable groups and reduce hospitalization [36].

Delivering high booster coverage requires secure planning on vaccine stock from the government's end. To provide almost half of West Java's population with booster shots, it would require the government to have at least 750,000 booster shots stock ready. Based on national data on vaccine stock, West Java's vaccine stock is less than 600,000 doses per early November 2022 [37]. Some of that stock might also be allocated for primary vaccination since there are still a few of West Java's citizens that have not received their primary vaccine doses. In addition to that, the types of vaccines in stock should also be highlighted in booster vaccination. The type of vaccine used for booster in regard to that of primary vaccines can be the same (homologous) or different (heterologous). A phase 4 immunogenicity study in Brazil found that heterologous boosting resulted in more robust immune responses compared to homologous boosting and that it might enhance protection [38]. A systematic review study also found that although the heterologous booster group had a higher risk of fever, myalgia, malaise, or fatigue within one week after receiving booster shots compared to the homologous booster group, heterologous booster has higher vaccine effectiveness compared to homologous booster in the prevention of SARS-CoV-2 infection, symptomatic COVID-19, and COVID-19 hospital admissions [39].

To maintain the quality of booster stocks, adequate and standardized storage facilities should be in place. Indonesia's vaccine cold chain system was established mainly to distribute vaccines for the national childhood immunization program, which only requires a temperature of 2–8° Celsius. This was expected to pose a limitation to effectively distributing COVID-19 vaccines, some of which require substantially lower temperature for transport, e.g. Pfizer and BioNTech which needs −70° Celsius [40]. A recent COVID-19 vaccine management evaluation further confirmed that only 31.5% of the assessed 517 public health centers in Indonesia have adequate vaccine storage capacity, while the rest of them lack refrigerator storage space [41]. This limitation should be paid attention to during booster vaccination rollout, especially with routine vaccination programs being reinstated that might share the already limited capacity of these cold chain facilities.

To ensure booster vaccination program implementation, ensuring sufficient healthcare workers is also crucial. During the pandemic, primary healthcare workers are already struggling with additional workload due to service disruption, which results in more complicated cases and workload [40]. As routine immunizations and more health services are getting restored, healthcare workers might experience similar struggles to balance multiple things. In Indonesia, it was reported that 83% of healthcare workers experienced moderate to severe burnout during the pandemic, and 41% were emotionally fatigued [1]. This is similar to the experience of healthcare workers in various parts of Africa that also experienced mental health issues during the pandemic due to stressful working conditions [42]. These points out the need to support and empower existing healthcare workers throughout COVID-19 responses, especially with a plan for an addition to the booster vaccination program.

To our knowledge, this is the first modelling study to investigate the need for additional COVID-19 booster initiatives in Indonesia that also illustrates a feasible vaccination time frame. However, this study has several limitations to note. First, the model did not take public health social measures (PHSM) such as non-pharmaceutical interventions and lockdown measures into account. Hence, the results of this study should be taken carefully, with consideration of PHSM implemented during the period when the data was collected and when the suggested booster vaccination would take place. Second, underreporting of COVID-19 cases is a constant issue throughout the pandemic, which might also affect the dataset used for this study. Therefore, overestimation or underestimation of predicted cases rooted from this particular reason might happen to our model. Lastly, this study did not take into account the number of people that have contracted COVID-19 multiple times. If many people have that condition, the population might be more immune to the disease than this study predicts, decreasing the target number of people that should be receiving booster vaccines in the examined period. Further study is warranted to validate the model using national data.

To implement this study's findings, some key factors should be confirmed beforehand. First, booster vaccine stocks should be ensured to be sufficient to meet the target. Prioritizing heterologous vaccine booster into the vaccination plan is highly recommended to enhance booster vaccine effectiveness. Second, adequate storage facilities and sufficient healthcare workers should be secured during the booster vaccination period. Insufficient resources can lead to prolonged vaccination program duration, missing the critical coverage and time-based target to impede future COVID-19 surges.

## 5 Conclusion

This paper presented the use of a mathematical model to aid local public health intervention in Indonesia. We successfully deployed a modified SEIR model that considers vaccine brands and the waning of vaccine-induced immunity, to help predict COVID-19 active cases in different booster coverage levels in a high COVID-19 burden area. This model was the most satisfactory among other methods, to accommodate both infectious diseases' epidemic patterns and related public health interventions. The result showed that a minimum of 70% booster coverage in West Java province is required to hamper future COVID-19 surge in the region, and our further assessment advised that such coverage should be achieved in early 2023 to achieve optimal public health outcomes. Proper vaccination planning should be in place to acquire such a target, which includes securing adequate vaccine supplies, storage facilities, and administering officers.

## Ethics statement

This is a modelling study based on COVID-19 statistics gathered by West Java's Provincial Health Office using de-identified data. Therefore, this study does not require ethical review and clearance.

## Author contribution statement

Nuning Nuraini; Bony Wiem Lestari: Conceived and designed the experiments; Contributed reagents, materials, analysis tools or data; Wrote the paper.

Almira Alifia; Kamal Khairudin Sukandar: Performed the experiments; Analyzed and interpreted the data; Contributed reagents, materials, analysis tools or data; Wrote the paper.

Fadiya Nadhilah Soekotjo: Analyzed and interpreted the data; Contributed reagents, materials, analysis tools or data.

## Data availability statement

Data will be made available on request.

## Declaration of competing interest

The authors declare that they have no known competing financial interests or personal relationships that could have appeared to influence the work reported in this paper.
