# Supplementary material for: Assessing potential surge of COVID-19 cases and the need for booster vaccine amid emerging SARS-CoV-2 variants in Indonesia: A modelling study from West Java
Source: Heliyon. 2023 Sep 9;9(9):e20009. doi: 10.1016/j.heliyon.2023.e20009 (PMC10559733; doi:10.1016/j.heliyon.2023.e20009)
Supplement: Multimedia component 1 [file mmc1.docx]

**Appendices**

**Appendix 1**

- 1. ***SEIR Model***

We constructed deterministic models that allow us to analyze the effect of different vaccine brands on a population that got exposed to more than one COVID-19 variant. To accommodate the effect of five vaccine brands and three groups of COVID-19 variants, we dealt with a system comprised of 60 variables in total. Each variable denotes the number of susceptible ($S$), exposed ($E$), infected ($I$), and recovered ($R$) individuals specific to its vaccine brand and COVID-19 variant. For example, $E_{v_{j}}^{i}$ denotes the number of individuals vaccinated with vaccine brand $j$ being exposed to COVID-19 variant $i$. The illustration of how each variable interacts with one another in the disease transmission flow is depicted in Figure 1.

The mathematical representation of the modified SEIR model is given below. Those with subscript $u$ represent the unvaccinated population, while subscript $v_{j}$ denotes the individuals with protection by vaccine brand $j$. Moreover, subscript $i$ refers to the infecting variant of COVID-19.

| $\frac{dS_{u}}{dt}=-\left( \sum_{i=1}^{n} \frac{\beta^{i}I_{u}^{i}}{N}+\sum_{i=1}^{n} \sum_{j=1}^{m} \left( 1-\rho_{S_{j}}^{i} \right)\beta^{i}\frac{I_{v_{j}}^{i}}{N}+\sum_{j=1}^{m} \theta_{j}v \right)S_{u}+\sum_{i=1}^{n} \delta_{u}R_{u}^{i}+\sum_{j=1}^{m} \tau S_{v_{j}}$ $\frac{dE_{u}^{i}}{dt}=\left( \sum_{i=1}^{n} \frac{\beta^{i}I_{u}^{i}}{N}+\sum_{i=1}^{n} \sum_{j=1}^{m} \left( 1-\rho_{S_{j}}^{i} \right)\beta^{i}\frac{I_{v_{j}}^{i}}{N} \right)S_{u}-\left( \sum_{j=1}^{m} \theta_{j}v+\eta\right)E_{u}^{i}$ $\frac{dI_{u}^{i}}{dt}=\eta E_{u}^{i}-\gamma I_{u}^{i}$ $\frac{dR_{u}^{i}}{dt}=\gamma I_{u}^{i}-\left( {\sum_{j=1}^{m} \theta_{j}v+\delta}_{u} \right)R_{u}^{i}$ $\frac{dS_{v_{j}}^{i}}{dt}=-\left( \sum_{i=1}^{n} \left( 1-\rho_{S_{j}}^{i} \right)\beta^{i}\frac{I_{u}^{i}}{N}+\sum_{i=1}^{n} \sum_{j=1}^{m} \left( 1-\rho_{S_{j}}^{i} \right)\beta^{i}\frac{I_{v_{j}}^{i}}{N}+\tau\right)S_{v_{j}}+\theta_{j}vS_{u}+\sum_{i=1}^{n} \delta_{v}R_{v_{j}}$ $\frac{dE_{v_{j}}^{i}}{dt}=\left( \left( 1-\rho_{S_{j}}^{i} \right)\beta^{i}\frac{I_{u}^{i}}{N}+\sum_{j=1}^{m} \left( 1-\rho_{S_{j}}^{i} \right)\beta^{i}\frac{I_{v_{j}}^{i}}{N} \right)S_{v_{j}}+\theta_{j}vE_{u}^{i}-\eta E_{v_{j}}^{i}$ $\frac{dI_{v_{j}}^{i}}{dt}=\left( 1-\rho_{E_{j}}^{i} \right)\eta E_{v_{j}}^{i}-\gamma I_{v_{j}}^{i}$ $\frac{dR_{v_{j}}^{i}}{dt}=\rho_{E_{j}}^{i}\eta E_{v_{j}}^{i}+\gamma I_{v_{j}}^{i}-\delta_{v}R_{v_{j}}^{i}+\theta_{j}vR_{u}^{i}$ | …(i) |
| --- | --- |

for $i=1,2,\ldots,n$ and $j=1,2,\ldots,m$, with $n$ and $m$ represent the number of COVID-19 variants and vaccine brands being observed, respectively. $N$ is the population size which comprises all state variables. All dynamic changes considered in this model are purely by the COVID-19 transmission, ignoring all aspects including natural birth and death. All parameters that existed in system 1 are briefly described in Table A1.1. While the rest of the parameters are assumed to be constant over time, the rate of vaccination $v,$and disease transmission $\beta$, are assumed to change over time. The detailed explanation regarding the calculation of vaccination rate is discussed later in subsection 1.2.

**Table A1.1**. Description of parameters used in SEIR model.

| Notation | Description | Values | Notes/References |
| --- | --- | --- | --- |
| $\beta^{i}=\beta^{i}(t)$ | Time-dependent transmission rate of COVID-19 variant $i$ | Estimated | Estimation details can be seen in Appendix 2.2 |
| $\rho_{S_{j}}^{i}$ | Efficacy of vaccine brand $j$ at preventing infections of variant $i$ | See Table A1.2 | (19) |
| $\rho_{E_{j}}^{i}$ | Efficacy of vaccine brand $j$ at preventing disease by variants $i$ | See Table A1.2 | (19) |
| $\theta_{j}$ | Proportion of vaccine brand $j$ delivered within population | See Figure A2.2 | (2) |
| $v=v(t)$ | Time-dependent vaccination rate | Estimated | Estimation details can be seen in Appendix 1.2 |
| $\delta_{u},\delta_{v}$ | Reinfection rate for unvaccinated and vaccinated individuals, respectively | 1/365 | (5,6) |
| $\tau$ | Vaccine-induced immunity waning rate | 1/365 | (4,6) |
| $\eta$ | COVID-19 incubation rate | 1/6 for all groups of variants | (20,21) |
| $\gamma$ | COVID-19 recovery rate | 1/14, 1/8 and 1/4 for the first, second, and third variant groups respectively | (21) |

**Table A1.2**. Vaccine effectiveness at preventing infection and severe disease in response to spread of three major group variants (19).

|  | Preventing infection | | | Preventing severe disease | | |
| --- | --- | --- | --- | --- | --- | --- |
|  | Group 1 | Group 2 | Group 3 | Group 1 | Group 2 | Group 3 |
| CoronaVax | 47% | 46% | 24% | 50% | 49% | 37% |
| AstraZeneca | 63% | 69% | 36% | 94% | 94% | 71% |
| Sinopharm | 68% | 67% | 35% | 73% | 71% | 53% |
| Moderna | 92% | 91% | 44% | 97% | 97% | 72% |
| Pfizer | 86% | 84% | 48% | 95% | 95% | 73% |

- 1. ***Vaccination Rate***

We used a standard logistic formula to depict the total vaccines administered in a population. The explicit formula of total vaccinated individuals at time $t$, denoted by $V(t)$, is given by

| $V\left( t \right)=\frac{fN}{(1+e^{-k\left( t-t_{0} \right)})}$ | …(ii) |
| --- | --- |

with $f$ is the vaccination coverage, ranging from 0 to 1. The other parameters, $k$ and $t_{0}$, represents the intrinsic growth and shifting factor, respectively. The rate of vaccination is simply the first derivation of this formula normalized relative to the population size. The explicit formula of the rate of vaccination that aims to inoculate $f$(in percent) of the total population is given by.

| $v_{0}\left( t \right)=\frac{fke^{-k\left( t-t_{0} \right)}}{\left( 1+e^{-k\left( t-t_{0} \right)} \right)^{2}}$ | …(iii) |
| --- | --- |

The retrospective vaccination rate, $v_{0}\left( t \right)$, follows a gaussian-like curve with a total area under the curve approximately $f$ and depicts the historical effect of vaccination.

**Appendix 2**

***2.1. Fitting Scheme for Vaccination Rate***

In this research, we have two deterministic models: [1] the modified SEIR model for the disease dynamics and [2] the rate of vaccine inoculation. For the rate of vaccination, we used the data of cumulative vaccines delivered in the population to yield the parameters of $k$ and $t_{0}$ from [2] with $N=48$ million and $f=0.8$, as the government initially planned to inoculate at least 80% of the population. To obtain the parameters, we implemented optimization that minimizes the cost function representing the root-mean-squared error between the model and the recorded data. Since the government has implemented two different independent vaccination schemes (primary and booster vaccinations), we estimated the parameters for each plan (see Figure A2.1 (A)), resulting in a multimodal vaccination rate (see Figure A2.1 (B)). In addition, we also considered the number and proportion of vaccine brands used in Indonesia, which is illustrated in Figure A2.2.


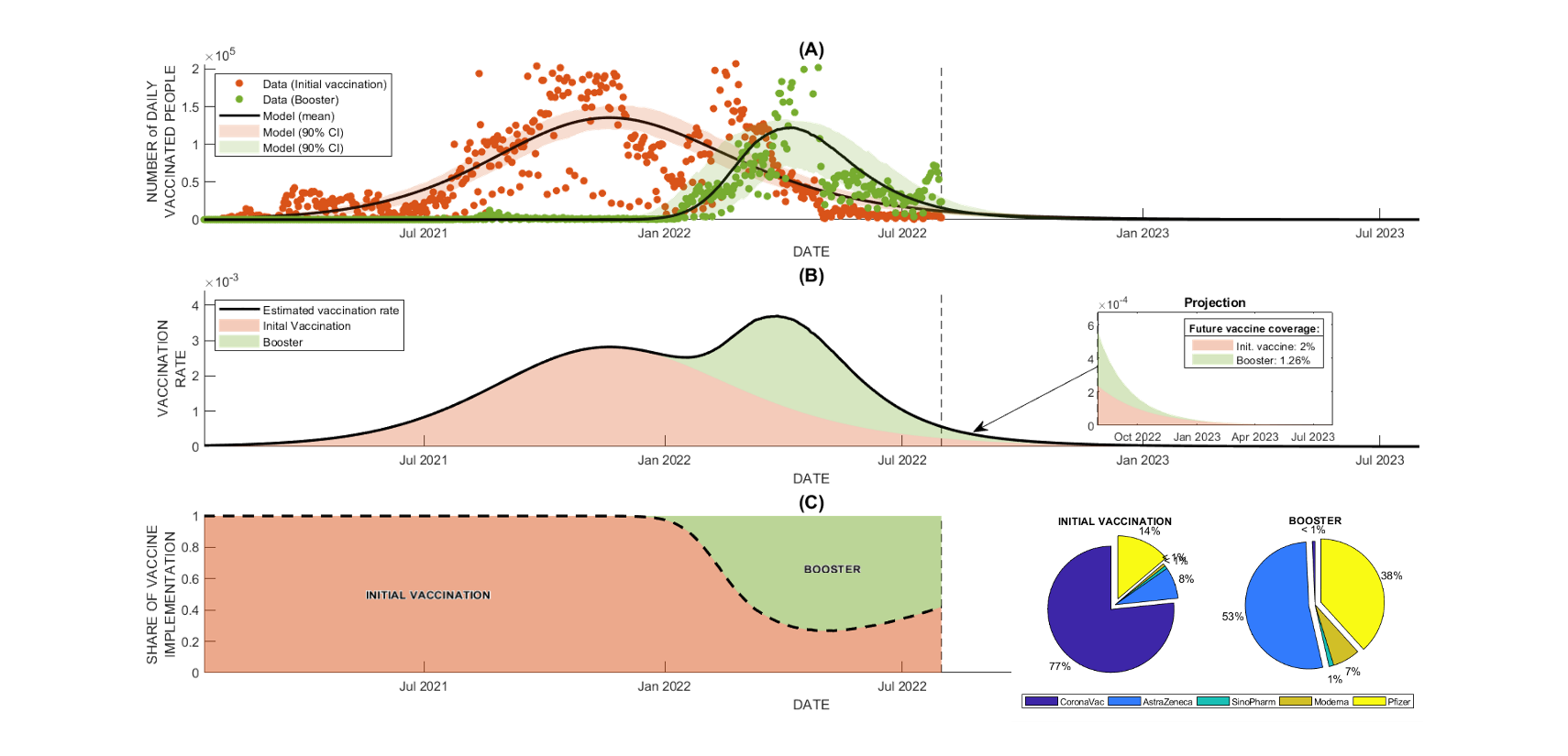


**Figure A2.1**. (A) Fitting results for the rate of vaccination (primary vaccinations in orange and booster plan in green). The confidence intervals shown in the figure were calculated using the MCMC method; (B) Multimodal vaccination rate, combining the vaccination rates for primary vaccinations (orange) and booster (green).


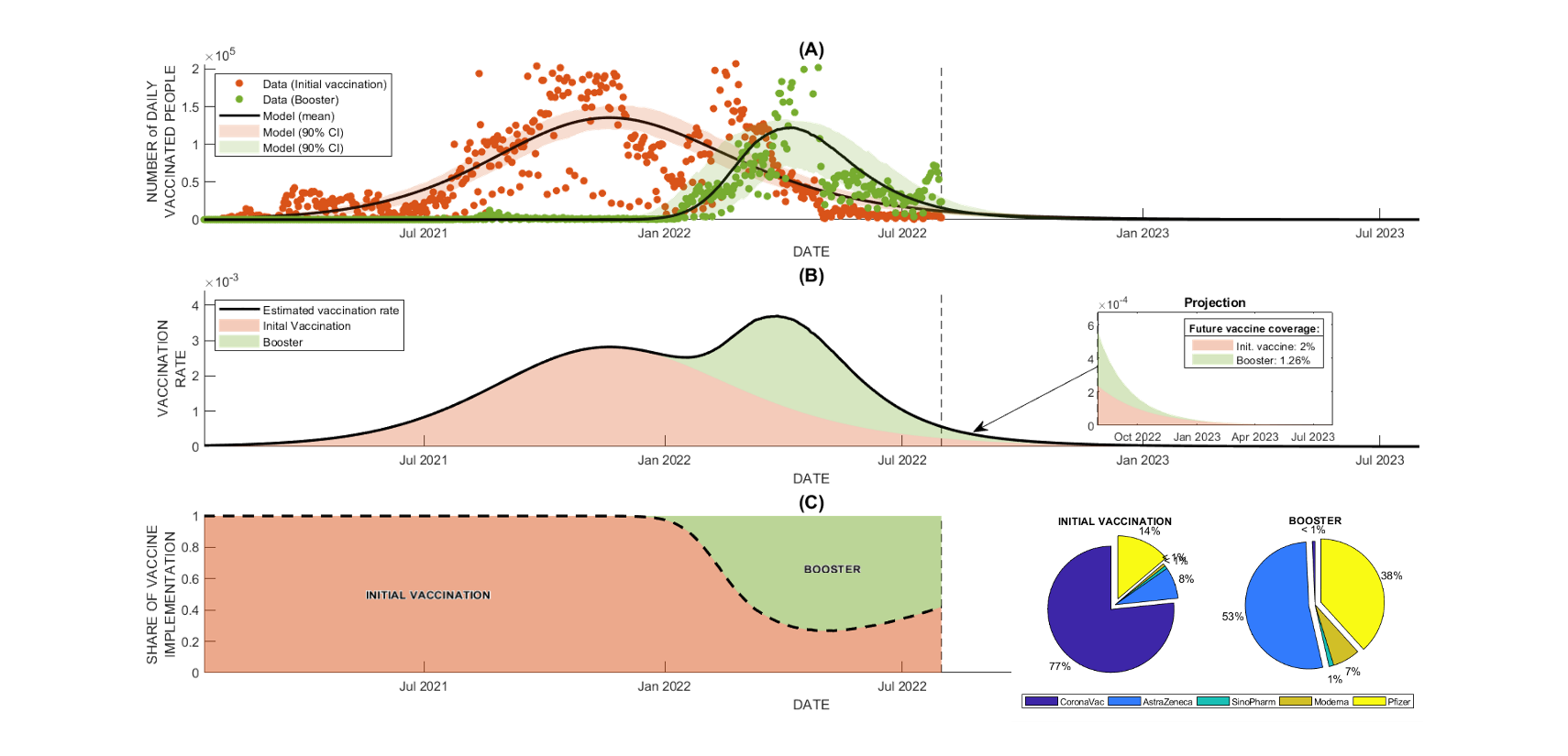


**Figure A2.2**. The proportion of vaccine brands implemented in West Java, Indonesia (2).

To conduct prediction of future COVID-19 cases, we defined $v_{1}(t)$ to describe prospective booster shots. The formula of $v_{1}(t)$ follows Equation (iii) with no parameter estimation involved but adjusting the vaccine coverage $f$ and the vaccination period to allow us on assessing the effect of such continued booster shots. Upon simulation, the rate of vaccination being used is the sum of both retrospective and prospective vaccination rates.

***2.2. Fitting Scheme for Transmission Rate***

The rate of transmission is the daily chance of susceptible individuals getting infected after making a contact with infected individuals. This quantity measures how massive the virus spread within the population. Since the transmissibility of the virus is strongly related to the general behaviour of its population, then this parameter is assumed to change over time. By considering $n$ as the number of COVID-19 variant groups, $\beta^{i}\left( t \right)$ denote the rate of transmission of COVID-19 variant $i$ at time $t$, which $i=1,2,\ldots,n$.

The transmission rate $\beta^{i}$ were estimated by searching for the best dynamics in which its rendered model fits the actual data of COVID-19 spread. The optimization problem is solved through the following notation:

$$\min_{\beta^{i}\mathcal{\in B}} \left( \sum_{k=1}^{K} \left( AC^{i}\left( t_{k} \right)-(I_{u}^{i}\left( t_{k},\beta^{i} \right)+\Sigma_{j}I_{v_{j}}^{i}\left( t_{k},\beta^{i} \right)) \right)^{2} \right)$$

$AC^{i}(t_{k})$ denotes the number of infections by variant $i$ at time-$tk$, while $K$ represents the length of the data. Since there were no detailed active cases data for each variant, we used the share of analysed sequences in Indonesia provided by Our World in Data. This data was used to derive the estimated active cases for each observed variant from the total active cases.

In the original SEIR model, the parameters including transmission rate are considered constants (17). However, since this study acknowledges three COVID-19 variant groups, the transmission rate parameter should be specified for each variant, notated as $\beta^{i}$, with $i$ indicating the variant group. Because the spread of each variant changes over time, we altered the transmission rate parameter to follow this time-dependent pattern. We split our time-series data into small chunks and fit them into the model with respective initial conditions. This allowed our model to have a time-dependent transmission rate that leads to a well-fitted model. The average values can then be used as a constant transmission rate for COVID-19 prediction.

Figure A2.3 (A)-(C) shows the estimated transmission rates for the three mentioned variant groups, while Figure A2.3 (D) depicts the model results compared to the observed data. The confidence intervals shown here are the implications of using the MCMC method to sample the unknown parameters from its estimated posteriors.


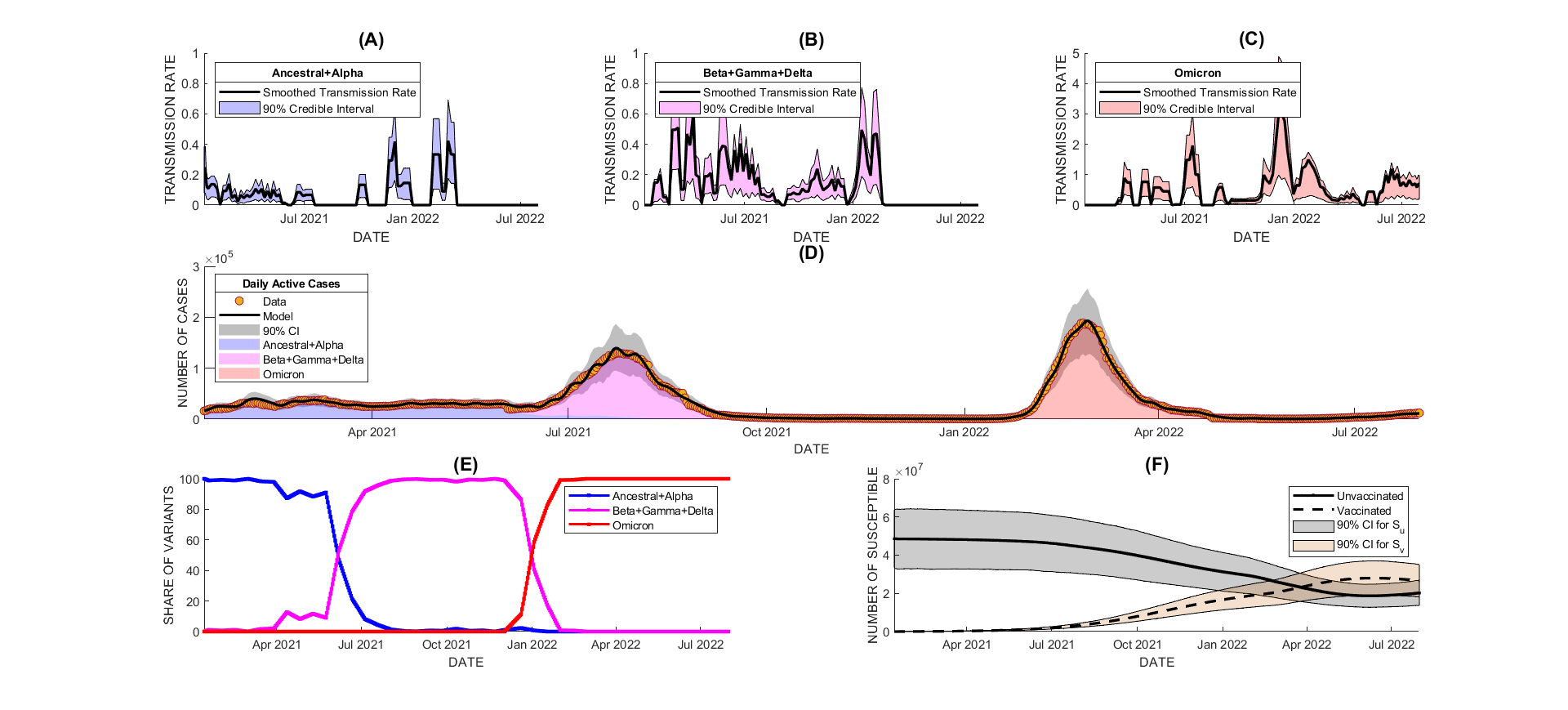


**Figure A2.3**. (A)-(C) Estimated transmission rates along with its 90%-confidence intervals; (D) Fitting results of the SEIR model with 3 groups of variants compared to the aggregated data of active cases; (E) The estimated share of variant groups, and (F) The estimated dynamics of susceptible individuals.

***2.3. MCMC method***

When estimating parameters for data fitting, it is common to use optimization methods to minimize the cost function so that the model fits the actual data. In this research, we used MCMC to do the task. The primary goal of this Bayesian inference is to evaluate the posterior densities of the unknown parameters given the set of observation data. From Bayes theorem, we have:

$$p\left( \theta| F^{obs}\left( i \right) \right)=\frac{p\left( F^{obs}\left( i \right) | \theta\right)\cdot p(\theta)}{normalizing constant}$$

Alternatively, we may state that the posterior is proportional to the prior times of the likelihood function. In a sense of data fitting, it is reasonable to assume:

$$F^{obs}\left( i \right)=F\left( i,\theta\right)+\epsilon_{i}$$

with $\epsilon_{i}$is normally distributed with zero mean and variance of $\sigma^{2}$. Hence, the likelihood function for this data fitting scheme becomes:

$$p\left( F^{obs}\left( i \right) | \theta\right)=\exp\left( -\frac{SSq}{2\sigma^{2}} \right)$$

where the $SSq$ stands for the sum-squared error formula that was described in the previous section. To evaluate the posterior densities, one of the methods is that introduced by Goodman and Weare in 2010 that computationally less expensive compared to directly evaluating the solution of the integral form (22). Once we obtained the estimated posterior densities of the targeted parameters, we sampled hundreds to thousands of points for the calculation of the confidence intervals.

**Appendix 3: Vaccine Rollout Assessment**

To evaluate the projected curve of COVID-19 dynamics, we averaged the estimated transmission rates to be used in the SEIR model with the initial conditions that conformed to the observed data. Regarding the effect of the vaccine, we set the prospective vaccine rates using (3) with various vaccine coverage f and fixed time delivery—to be finished in early 2023. This setting can be seen in Figure 6(A)-(D) with the hypothetical additional vaccination functions shown in dashed green. Adding the corresponding additional vaccination rataes to the SEIR model resulted in the projected COVID-19 dynamics given in Figure 2 in the main manuscript.


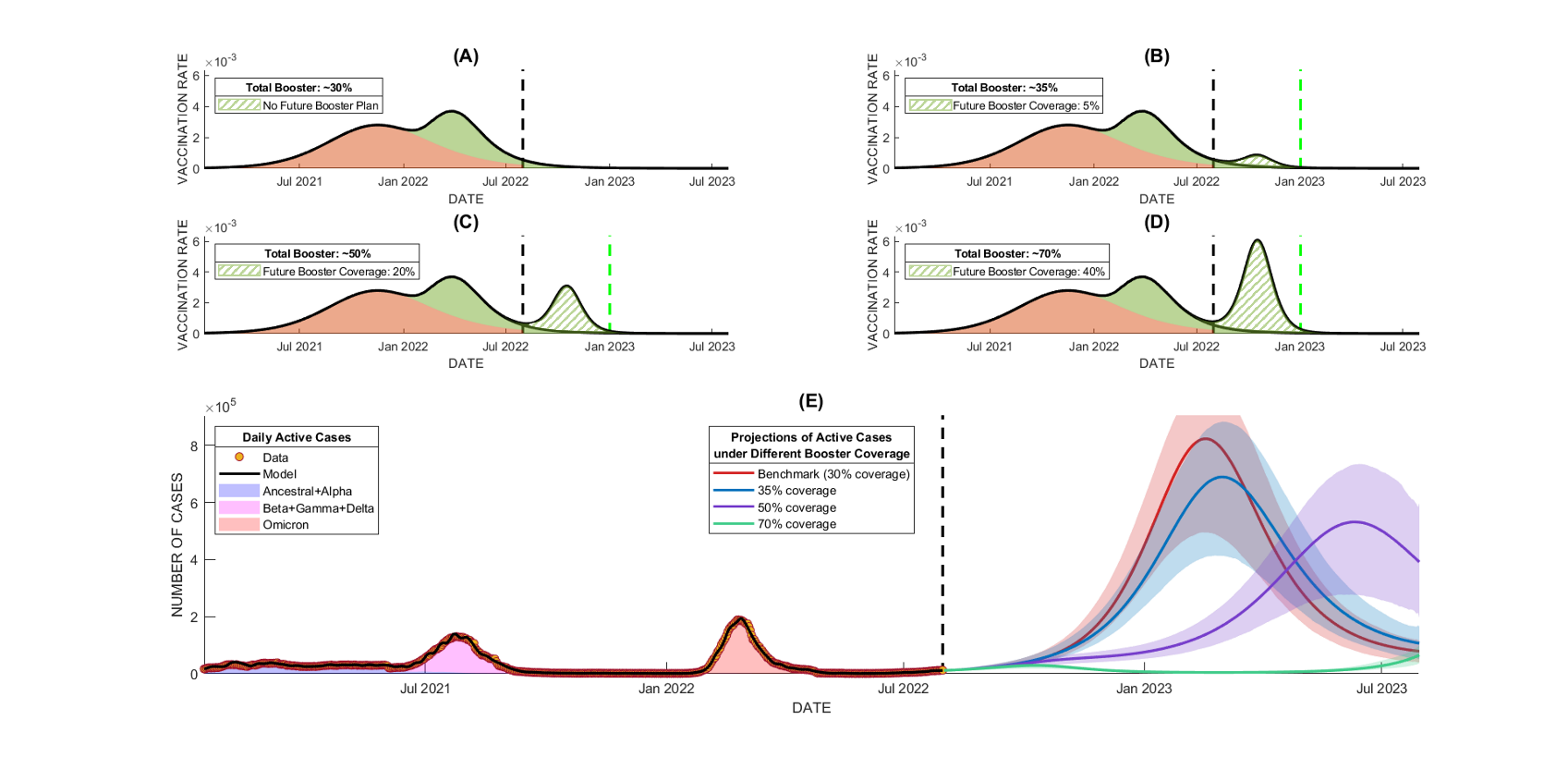


**Figure A3.1**. Four scenarios by varying vaccine coverages (A) benchmark (no further booster plan); (B) additional 5%; (C) additional 20%; (D) additional 40% of booster plan, set to be finished in early 2023.

Furthermore, we extended the vaccine rollout assessment to accommodate variations in vaccine coverage and the time or period of vaccine delivery. While varying the vaccine coverage is explicitly made by varying the value of f, setting the time of vaccine delivery is more challenging. Recall the formula of vaccination rate with coverage $f$, growth rate $k,$ and its shifting factor of $t_{0}$that follows Equation (3). If, $T_{0}$ and $T_{A}$ are the starting and ending points of the additional booster shots, then $T_{A}-T_{0}$ is the period of vaccine delivery. Mathematically, these two conditions hold for a small $\zeta$:

1. When $t=T_{0}$, we have $V\left( T_{0} \right)=\zeta fN\Rightarrow\left( 1+e^{-k\left( T_{0}-t_{0} \right)} \right)=1/\zeta$, and
2. When $t=T_{A}$, we have $V\left( T_{A} \right)=\left( 1-\zeta\right)fN\Rightarrow\left( 1+e^{-k\left( T_{A}-t_{0} \right)} \right)=1/(1-\zeta)$

Given $T_{0}$ and $T_{A}$, we can solve the two equations to give us the appropriate values of $k$and $t_{0}$ for the additional vaccination rates with adjustable vaccine coverage $f$ and time of vaccine delivery. Figure 3 in the main manuscript shows the predicted fraction of total cases estimated using the SEIR model by varying both vaccine coverage $f$ and time delivery.
